# Sex Differences in Cognitive Flexibility and Resting Brain Networks in Middle-Aged Marmosets

**DOI:** 10.1523/ENEURO.0154-19.2019

**Published:** 2019-07-25

**Authors:** M. LaClair, M. Febo, B. Nephew, N.J. Gervais, G. Poirier, K. Workman, S. Chumachenko, L. Payne, M.C. Moore, J.A. King, A. Lacreuse

**Affiliations:** 1 Neuroscience and Behavior Program, University of Massachusetts, Amherst, MA 01003; 2 Department of Psychology, Fairfield University, Fairfield, CT 06824; 3 Department of Psychiatry, University of Florida, Gainsville, FL 32610; 4 Worcester Polytechnic Institute, University of Massachusetts Medical School, Worcester, MA 01655; 5 Center for Neuroendocrine Studies, University of Massachusetts, Amherst, MA 01003; 6 Psychological and Brain Sciences, University of Massachusetts, Amherst, MA 01003; 7 Center for Comparative Neuroimaging, University of Massachusetts Medical School, Worcester, MA 01655

**Keywords:** animal models, cognitive flexibility, executive function, resting state functional connectivity, reversal learning, sex differences

## Abstract

Sex differences in human cognitive performance are well characterized. However, the neural correlates of these differences remain elusive. This issue may be clarified using nonhuman primates, for which sociocultural influences are minimized. We used the marmoset (*Callithrix jacchus*) to investigate sex differences in two aspects of executive function: reversal learning and intradimensional/extradimensional (ID/ED) set shifting. Stress reactivity and motor function were also assessed. In agreement with human literature, females needed more trials than males to acquire the reversals. No sex differences in ED set shifting or motivational measures were observed. The findings suggest enhanced habit formation in females, perhaps due to striatal estrogenic effects. Both sexes showed increased urinary cortisol during social separation stressor, but females showed an earlier increase in cortisol and a greater increase in agitated locomotion, possibly indicating enhanced stress reactivity. Independent of sex, basal cortisol predicted cognitive performance. No sex differences were found in motor performance. Associations between brain networks and reversal learning performance were investigated using resting state fMRI. Resting state functional connectivity (rsFC) analyses revealed sex differences in cognitive networks, with differences in overall neural network metrics and specific regions, including the prefrontal cortex, caudate, putamen, and nucleus accumbens. Correlations between cognitive flexibility and neural connectivity indicate that sex differences in cognitive flexibility are related to sex-dependent patterns of resting brain networks. Overall, our findings reveal sex differences in reversal learning, brain networks, and their relationship in the marmoset, positioning this species as an excellent model to investigate the biological basis of cognitive sex differences.

## Significance Statement

We examined sex differences in multiple outcomes [cognition, motor function, stress reactivity, and resting state functional connectivity (rsFC)] in middle-aged marmosets. We found that female marmosets had poorer reversal learning relative to males. rsFC analyses revealed substantial sex differences in cognitive networks, with differences in both overall neural network metrics and specific regions, including the prefrontal cortex, caudate, putamen, and nucleus accumbens. Sex-dependent correlations between reversal learning and neural connectivity measures indicate that the sex difference in cognitive performance is related to sex-dependent patterns of resting brain networks. Although these data are correlational and cannot determine causal effects, they are consistent with human resting state data, supporting the idea that cognitive sex differences have identifiable intrinsic neural correlates.

## Introduction

Sex and gender differences in cognitive performance are an actively debated topic, both in the public domain and the scientific community. The American Community Survey performed by the United States Census Bureau in 2011 found underrepresentation of women in science, technology, engineering, and math (STEM) fields, with men representing 74% and women 26% of STEM workers, despite women outperforming men overall in higher education (Flashman, 2013). With increased focus on gender disparities in STEM comes greater interest in the biological factors that impact sex-based differences in cognitive performance.

In humans, sex differences in certain cognitive domains are well established (Kimura, 1992; Halpern, 2000; Hampson, 2002). Men outperform women on many spatial tasks, including mental spatial rotation (Voyer et al., 1995), while women outperform men on verbal tasks such as verbal fluency (Heinzel et al., 2013) and verbal memory (Munro et al., 2012; Murre et al., 2013). However, sex differences in cognitive performance may not be as clear-cut as previous studies may have suggested, as culture and gender stereotypes are known to impact performance on selective cognitive measures (Levine et al., 2005; Lippa et al., 2010; Miller and Halpern, 2014). Thus, sociological and biological influences are tightly intertwined in humans, highlighting the importance of studying appropriate animal models to understand the biological basis of cognitive sex differences.

We examined cognitive sex differences in the marmoset, a small (300 − 500 g) New World primate that is emerging as an attractive new primate model for neuroscience research (Okano et al., 2012; Miller, 2017; Prins et al., 2017). Marmosets share with other primates a complex brain and behavior but, because of their small size, present a number of practical advantages as laboratory animals. They also have a relatively short lifespan of ∼10 years, which is advantageous for developmental research. The marmoset has a lissencephalic brain approximately five times larger than the rat brain. Although it is 12 times smaller than the rhesus monkey brain and 180 times smaller than the human brain (Solomon and Rosa, 2014), brain organization is well conserved among the three primate species (Chaplin et al., 2013). Marmosets can successfully perform a range of prefrontal-dependent tasks (Miles, 1957; Ridley et al., 1981; Lacreuse et al., 2014; LaClair and Lacreuse, 2016) and hippocampal-dependent tasks (Lacreuse et al., 2014). Little is known about sex differences in cognition in this species. One study conducted in 35 young marmosets (one to four years old) found no sex difference in the performance of visual discriminations and reversal tasks (Takemoto et al., 2015).

The second goal of this research was to investigate the neural correlates of cognitive sex differences in the marmoset using resting state functional connectivity (rsFC) with fMRI. rsFC exploits the fMRI signal to characterize temporally correlated fluctuations in neuronal activity when subjects are at rest. In humans, variations in rsFC have been associated with differences in cognitive performance (Sala-Llonch et al., 2012; Zou et al., 2013). In addition, sex differences in cognition have been reported to be associated with sex differences in brain networks (Ingalhalikar et al., 2014; Satterthwaite et al., 2015; Tunç et al., 2016). Marmosets can be trained to undergo conscious neuroimaging with minimal acclimation (Liu et al., 2013). Several studies have revealed four higher-order functional connectivity networks in marmosets that are similar to those found in humans (Belcher et al., 2013, 2016); however, the effects of sex on functional connectivity have not yet been examined in this species.

As in prior marmoset resting state fMRI studies (Liu et al., 2019) our objective was two-fold: (1) determine the organization of global functional connectivity in male and female marmosets, and (2) determine the relationship between cognitive behaviors and metrics reflecting functional connectivity patterns brain-wide. Our rationale for this approach was that cognitive behaviors investigated here emerge from neural activity and functional interactions of many distributed brain areas. Using network science metrics offers a unique way to test this hypothesis, which involves a broad number of brain regions. However, we should note that we did not have a priori predictions on the role of specific regions of interest (ROIs) in the orbitofrontal cortex (OFC)/striatum.

The present study compared the performance of middle-aged male and female marmosets (approximately five years of age) on tasks of executive function, including cognitive flexibility and attentional set shifting. To obtain a comprehensive understanding of factors impacting cognitive performance, we also investigated sex differences in motor performance and stress reactivity. Following behavioral tasks, monkeys were scanned for rsFC to investigate sex differences in brain networks and their potential relationship to cognitive function.

## Materials and Methods

### Subjects

Twenty-eight marmosets ranging from four to six years old were used for this study (14 females, mean age = 4.81 years; 14 males, mean age = 5.10). All marmosets were housed in male/female pairs at the University of Massachusetts, Amherst and maintained under a 12/12 h light/dark cycle (lights on at 7:30 A.M.) at an ambient temperature of 80 F with a relative humidity of 50%. The pairs were housed in steel mesh cages (101 × 76.2 × 78.7 cm) equipped with perches, hammock, nest boxes, and branches to encourage species-typical behaviors. Male marmosets were vasectomized in adulthood, before the start of the study, to avoid pregnancy. The characteristics of the marmosets and the tests they performed can be seen in Table 1. The monkeys were fed a daily diet of fresh food including fruits, vegetables, nuts and seeds, various breads, and ZuPreem marmoset food. Fruit and nuts were provided twice daily up until 2 h before and immediately after cognitive testing and water was available *ad libitum*. The monkeys were provided with daily enrichment, including foraging tubes and a variety of toys. The animals were cared for in accordance with the guidelines published in the Guide for the Care and Use of Laboratory Animals, 8th edition (2011). The studies were approved by the Institutional Animal Care and Use Committee of the University of Massachusetts Amherst and the University of Massachusetts Medical School, Worcester.

**Table 1.** Characteristics of study subjects: sex, date of birth, and age at test

Animal ID	Sex	DOB	Age at start of cognitive testing	Age at social separation	Age at motor testing	Age at rsFC imaging
02	Female	9/16/10	5.82	5.52	5.49	6.52
04	Female	9/16/10	5.32	5.59	5.41	6.56
06	Female	7/5/11	4.93	4.67	4.68	5.64
08	Female	1/4/10	6.05	6.16	6.09	7.32
10	Female	7/5/11	4.52	4.81	N/A	5.93
12	Female	3/22/11	4.82	5.01	4.95	5.76
14	Female	11/23/11	4.12	4.36	4.26	N/A
15	Female	1/18/11	4.99	5.21	5.14	6.45
17	Female	4/2/12	4.21	4.01	4.07	5.53
19	Female	1/6/12	4.78	4.25	4.35	N/A
21	Female	11/18/11	4.72	4.71	4.47	5.61
23	Female	4/28/12	4.08	3.96	3.96	5.13
26	Female	3/18/12	4.41	4.34	4.44	5.70
28	Female	3/28/12	4.55	4.34	N/A	N/A
01	Male	6/1/11	4.64	4.87	4.78	5.82
03	Male	6/18/10	5.56	5.73	5.72	6.81
05	Male	5/1/11	4.69	4.86	4.84	5.81
07	Male	9/3/09	6.86	6.51	6.48	7.65
09	Male	8/20/10	5.52	5.57	5.52	6.80
11	Male	10/28/10	5.21	5.39	5.27	6.15
13	Male	11/28/11	4.65	4.64	N/A	N/A
16	Male	5/13/12	3.96	3.92	N/A	5.13
18	Male	5/10/11	5.30	4.93	5.01	6.42
20	Male	4/8/11	5.21	5.04	5.05	N/A
22	Male	6/4/11	5.01	4.88	4.93	6.07
24	Male	11/9/11	4.77	4.41	4.42	5.59
25	Male	8/4/11	N/A	5.06	N/A	N/A
27	Male	9/28/11	5.03	4.81	4.92	6.17

N/A indicates animal did not complete test.

### General procedure

Monkeys received comprehensive assessments of cognitive function, stress reactivity and motor function. The details regarding each assessment are provided below. Monkeys were trained on cognitive tasks 5 d per week, with training spanning several months. Tests of motor function were conducted concurrently at times when the monkeys were not engaged in cognitive testing. The social separation task was conducted on a single day during which monkeys were not engaged in any other task.

### Cognitive assessments

Monkeys were tested on the Cambridge Neuropsychological Test Automated Battery (CANTAB), an automated cognitive testing battery used with humans (Robbins et al., 1994), and nonhuman primates (NHPs), including marmosets (Roberts et al., 1988; Spinelli et al., 2004).

#### Testing apparatus

The nonhuman primate version of the CANTAB (Monkey CANTAB Intellistation with Liquid Reward, Model 80951A) consisted of a touch screen panel (37.78 cm) in a stainless-steel frame (56 × 38 × 30 cm) using an Intel-based 1.6-GHz CPU operating system. A stainless-steel sipper tube in the middle of the screen delivered the reward (banana milkshake) via a peristaltic pump, at a rate of 0.2 ml/s.

#### Procedure

To encourage participation, food and water were removed from the animals’ cages 2 h before testing and replaced in the cage no later than 5 h after removal. For testing, marmosets voluntarily entered a transport box (34.1 × 20.65 × 30.8 cm) made of clear Plexiglas attached to the front of their homecage. This set-up allowed the focal animal to have visual, auditory, and olfactory access to their partner, as well as other animals in the colony. The CANTAB was positioned against the meshed front (2.5 × 2.5 cm openings) of the transport box, so animals could reach through to touch the screen and lick the reward from the sipper tube. Experimenters loaded CANTAB testing programs remotely from a desktop computer located outside of the marmoset housing rooms.

#### CANTAB training

We followed the procedures described by Roberts et al. (1988) and Pearce et al. (1998) for stages of tone-reinforcement associations and touch-training. Monkeys were trained to lick the milkshake from the spout, to associate a tone (41 Hz) with reward delivery (5 s), to touch the screen, touch a large static square at the center of the screen and touch smaller squares appearing successively at random locations on the screen, before being presented with the first pair of stimuli.

#### Simple reversal (SR) learning

**Figure 1.**
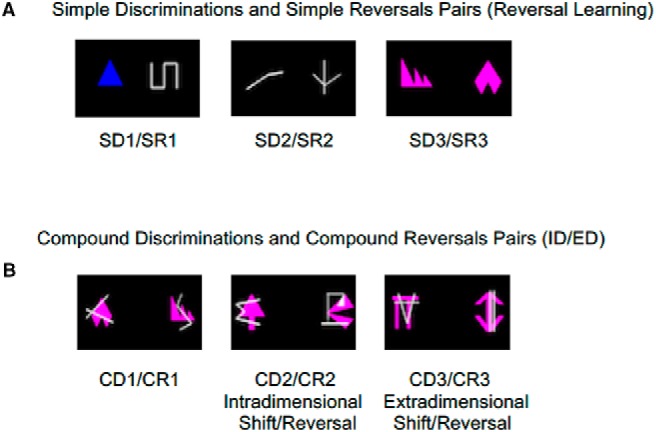
Examples of stimuli from SR learning (***A***) and from the ID/ED (***B***).

The marmosets were presented with a total of three pairs of stimuli depicted in Figure 1*A*. The first pair of stimuli consisted of a blue triangle and a white line. The second pair consisted either of two different white lines or two different pink shapes (the order of presentation of pairs 2 and 3 was counterbalanced between monkeys). For each pair, monkeys had to perform a simple discrimination (SD), followed by a SR. The two stimuli appeared in any position on the touch screen. Animals began with SD, pair 1 and were given a total of 40 trials a day, 5 d a week, to learn the stimulus/reward contingencies (for example, blue triangle always rewarded). Once animals reached a 90% correct criterion out of 40 consecutive trials, the stimulus/reward contingencies were reversed (e.g., the white line was now rewarded; SR pair 1). When the 90% accuracy criterion was reached on the SR, the marmoset moved on to a new stimulus pair (i.e., SD pair 2). The number of trials to reach the 90% learning criterion (TTC) were recorded, with fewer trials reflecting faster learning of the stimulus/reward contingencies. In addition, the number of omissions (trials on which the monkey made no choice) and the response times (RTs) on each trial were recorded as an index of motivation. To facilitate analyses between reversal learning performance and rsFC, a composite of performance, the reversal index (RI) was computed for each monkey by dividing the mean TTC across the three reversals (SR1, SR2, SR3) by the mean TTC on the three SDs (SD1, SD2, SD3). This composite score reflected how many more trials the monkey had needed to perform the reversals relative to pre-reversal discriminations, with a higher ratio reflecting poorer performance. As noted early by Rumbaugh and Jeeves (1966) such a ratio provides a better measure of reversal learning per se, by overcoming individual differences (in perception, anxiety, motor function) in discrimination abilities.

#### Intradimensional/extradimensional (ID/ED) set shifting

For the ID/ED, the marmosets were again presented with a total of three pairs of stimuli, however, each was a compound stimulus, consisting of a shape dimension and a line dimension, overlaid on top of one another (Fig. 1*B*). For the first discrimination (CD1) animals were given the exact same stimulus/reward contingencies as SR3 (e.g., same shapes and same rewarded stimulus as SR3), but with the addition of an extraneous dimension (lines) that they needed to ignore. It was followed by a reversal (CR1) in which they had to select the alternate shape of the pair. Animals were given 40 trials per day and were required to reach a 90% accuracy criterion to move on to the next stage of testing.

#### Intradimensional shift (pair 2)

the second pair of stimuli was made from new stimuli, but the target dimension (e.g., shape) from CD1/CR1 continued to apply and the other dimension (lines) was to be ignored.

#### Extradimensional shift (pair 3)

the final pair were new stimuli, but this time monkeys had to switch from using the previous target dimension (e.g., shape), to using the alternate dimension (e.g., line) as the target.

#### Statistical analysis

For SR learning, the TTC, errors to criterion, omissions, RT, and RI were analyzed using a mixed ANOVA with sex as a between-subjects factor and pair number (pair 1, pair 2, pair 3) and test type (SD, SR) as within-subjects factors. The same analysis was used for the ID/ED shifting task, with the TTC, errors to criterion, omissions, and RTs as variables.

### Social separation

In NHPs that form social bonds, mate separation can trigger robust hypothalamic-pituitary-adrenals (HPA) activation and behavioral indications of stress (Cross et al., 2004). Social separation has been used as a stressor in a number of NHP species, including the marmoset. We used the procedure described by French et al. (2007). Following urine collection (see Urine collection and assays section), the focal animal entered a transport box and was immediately transferred from its colony room to a similar cage in an empty room. Separated animals had access to food and water *ad libitum*. Animals were reunited with their cage mates at 15:30, after 7 h had elapsed. No cognitive testing occurred for the animal undergoing social separation on the day of separation.

#### Behavioral observations

Behavior was video-recorded with a SONY Handycam (HDD 2000× digital zoom) video camera provided with 0.45× wide angle lens. Animals were video-recorded at baseline (BL, 30 min before separation, 8:30 A.M.), throughout the separation period (Sep), and 24 h post-separation (Post-Sep; the day following separation, 8:30 A.M.). Behavior was scored from the video recordings using an interval schedule of 15 s, with behaviors occurring 0 − 20 times within a 5-min behavioral sample. All experimenters achieved 90% interrater reliability on behavioral observations before scoring videos. Behaviors included measures of locomotion, sociality, and aggression, adapted from an extensive ethogram developed for the marmoset (Stevenson, 1977). Behavior during the separation was scored as follows: t1 (first 5 min after experimenter left separation room), t2 (5-min sample 3.5 h after start of separation), and t3 (final 5-min sample of the 7-h separation). Behaviors from the three time points were then averaged to create an overall separation score.

#### Urine collection and assays

Urine samples were collected to assess cortisol levels in each animal immediately before separation (BL) and the morning following separation (Post-Sep), using a method described by Saltzman et al. (2004). Briefly, animals entered the transport box at 7:30, a few minutes after the lights turned on, and remained there until they urinated or until 30 min had elapsed. During the 7-h separation, experimenters entered the separation room once each hour and collected any available urine from a catch pan underneath the animal’s cage. Urine was pipetted into 1.5-ml vials, spun for 5 min and then frozen at −20°C. The Endocrine BioServices Assay Lab at the University of Nebraska Omaha, performed all urinary cortisol assays. Because of the variability in urine availability, urine samples were grouped as follows: averaged samples from hours 1 and 2 of separation (onset), averaged samples from hours 3, 4, and 5 (mid), and averaged samples from hours 5 and 6 (end). However, because too few urine samples were available in the first 2 h of the separation period, only the samples from the mid and end periods were included in the analyses.

#### Statistical analysis

Behavior and urinary cortisol were analyzed using a mixed ANOVA with sex as a between-subjects factor and test phase as a within-subjects factor. The analysis of behaviors focused on locomotor behaviors, as locomotion is a useful indicator of stress, previously shown to be altered by social separation in marmosets (Johnson et al., 1996). We recorded instances of agitated locomotion (defined as an animal moving more than one step in a directed plane, with an exaggerated gait, sometimes accompanied by piloerection, with tail extended or arched) and calm locomotion (defined as animal moving more than one step in a directed plane with a relaxed gait), with increased agitated locomotion being indicative of increased stress (Badihi et al., 2008). Correlations between behavior and change in cortisol from baseline to end (change = end-BL) were also performed.

### Motor task

**Figure 2.**
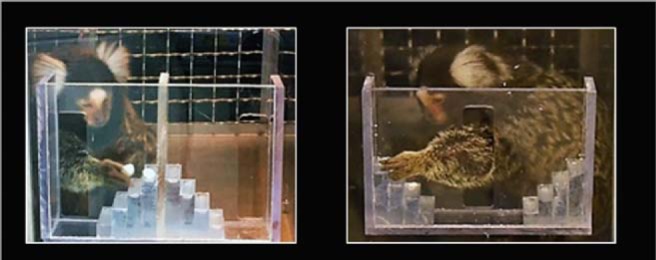
Picture of the hill and valley apparatus.

To assess motor function, we used the hill and valley task, a measure of fine motor ability that has previously been used in marmosets, especially in models of stroke and Parkinson’s disease (Eslamboli et al., 2003; Marshall et al., 2003; Bihel et al., 2010; Phillips et al., 2017). It assesses motor function in each limb as well as perceptual spatial impairment. The monkeys were tested in their housing room. As for the cognitive tasks, monkeys voluntarily entered a transport box attached to their home cage to access the hill or valley apparatus, securely attached to the front of the box via a Plexiglas screen (Fig. 2). Each apparatus had two five-step (9 × 9 × 3 mm) staircases, either rising away from a central opening (valley), or from two lateral openings (hill). The monkeys had to reach through these openings, using either their right or left hand, to retrieve one of the mini dehydrated marshmallows (6 mm in diameter) placed in the middle of each step. In the valley version, the central vertical slot (7.7 × 2 cm) allowed the marmoset to use its left hand to reach the reward located on its right, or the right hand to reach the reward located on its left (contralateral hemifield to hand used). In the hill version, entry was through two lateral slots (7.4 × 2 cm) on the side of each stair so that the monkey had to use its right hand to retrieve the rewards on the right stairs and the left hand to retrieve the rewards on the left stairs (ipsilateral hemifield to hand used).

Marmosets were trained on the hill and valley apparatus until they successfully retrieved a marshmallow from each step with each hand. If the marmoset failed to perform the task after 10 attempts, it was excluded from the task. For testing, marmosets were given a maximum of 5 min to retrieve all five marshmallows from one staircase of the apparatus. Each marmoset received four conditions (hill left, hill right, valley left, valley right) per session, one session per day, and performed a total of three testing sessions. The order of the hill and valley conditions was randomized (half received hill first, half valley first) and alternated each test day. If the marmoset failed to retrieve the five marshmallows within the 5-min time limit, the test session was rerun the following day. Marmosets received one point for retrieving the marshmallow on the first step, two points for retrieving from the second step, and so on, for a maximal accuracy score of 15 points per hand. Marmosets lost one point each time a marshmallow was dropped. The time to retrieval from the first reaching through the opening until retrieval of the last marshmallow was recorded for each condition.

#### Hand preference

Because hand preference had the potential to affect the ability of each hand, we determined the hand preference of each marmoset using a simple hand reaching task. Monkeys performed 50 reaches through the central slot of the valley apparatus to reach a mini marshmallow placed 7.7 × 2 cm from the slot. The number of left and right hand reaches was recorded. Any trials in which the marmoset used both hands were excluded. For each subject, a handedness index (HI) was determined by subtracting the number of left-handed responses from the number of right-handed responses and dividing by the total number of responses (Hopkins, 1999). HI values ranged from −1.0 to 1.0, with the absolute value representing the strength of the preference. The positive values indicated a right-hand bias while the negative values indicated a left-hand bias.

#### Statistical analysis

Mixed measures ANOVAs were performed on the Time to retrieval and accuracy scores, with sex as between-subjects factor and hand used as a within-subjects factor, and HI as a covariate.

### rsFC

We used the state-of-the-art technique developed by Dr. Afonso Silva (Silva et al., 2011), to image awake marmosets without the use of anesthetic. Each animal wore a sleeveless jacket (Lomir Biomedical, Inc), which attached to a semi-cylindrical plastic cover made of Lexan, restricting anterior or posterior movement but allowing the animal to move its arms, legs, and tail freely. The plastic cover was attached to the back of the marmoset’s jacket using plastic cable ties. The monkey laid in a prone, sphinx position, in the MRI bed, which consisted of a 111-mm cylindrical tube. The cover was secured to the bed by screwing nylon thumb screws into the bars on the bed. Each marmoset wore an individualized helmet adapted to their skull to support the head and prevent movement while providing comfort.

#### Acclimation

Before imaging sessions, animals were acclimated to the bed restraint device, noise related to imaging, and the helmet, following the procedures detailed in Silva et al. (2011). The entire acclimation period took between four and six weeks for each animal, with acclimation occurring 4–5 d a week.

#### fMRI data acquisition

The monkeys were scanned at the Center for Comparative Neuroimaging at the University of Massachusetts Medical School. Following 1 h acclimation to the neuroimaging room, marmosets were placed in jackets, arranged in the imaging cradle, positioned in the MRI bed, and imaged using a custom head coil as described in Silva et al. (2011). Imaging was conducted on a high-field MRI system. The system incorporated a 4.7 T/40 cm horizontal magnet (Oxford) equipped with 450 mT/m magnetic field gradients and a 20-G/cm magnetic field gradient insert (inner diameter = 11.5 cm; Bruker) with a digital interface to Bruker console, run by Paravision 6. Field map measurements allowed the estimation of the magnetic field inhomogeneity and shimming. For each marmoset, anatomic images were obtained using rapid acquisition relaxation enhanced (T2 Turbo RARE) sequence with relaxation time (TR) = 2892.968 ms, RARE factor = 8, echo time (TE) = 36 ms, resolution matrix = 256 × 256, field of view (FOV) = 45 mm × 45 mm, slice number = 25, slice thickness = 1.1 mm. Functional images were acquired using echo-planar imaging (EPI) with the same FOV and slice thickness, TR = 1691.038 ms, TE = 26.523 ms, flip angle = 90°, and resolution matrix = 128 × 128, for 11.27 min (400 repetitions). All monkeys were scanned within three months of cognitive testing.

#### rsFC image processing

Brain masks were first generated using FMRIB Software Library’s (FSL) Brain Extraction Tool (BET; Smith, 2002) on anatomic scans and masks were then manually adjusted with the help of ITKSNAP (www.itksnap.org). The masks outlining the brain were used to remove non-brain voxels. The cropped brain images were aligned with a Marmoset brain template (Liu et al., 2018) using the FSL linear registration program *FLIRT* (Jenkinson et al., 2002). Registration matrices for each subject were saved and used to subsequently transform functional datasets into atlas space for preprocessing and analysis. Aside from subject-to-atlas registration, which used FSL FLIRT, postprocessing steps were conducted using Analysis of Functional NeuroImages (AFNI; Cox, 1996). AFNI’s 3dDespike was used to remove time series spikes and this was followed by slice timing correction using 3dTshift. Motion correction was conducted using 3dvolreg, after which functional scans were aligned with the Marmoset template using FLIRT. Time series from motion estimates and from areas with CSF (CSF ventricles) and white matter were used as regressors. AFNI’s 3dTproject was used for the removal of motion-related, CSF and white matter signals, spatial blurring (0.8 mm FWHM), and whole-brain voxel-wise bandpass filtering between 0.01 and 0.1 Hz.

Time series fMRI signals from postprocessed scans were extracted from each ROI based on the atlas-guided seed location (122 bilateral placed seed regions included for 244 total ROIs). Time series for each voxel were averaged per ROI seed, exported as text files, and used voxel-wise cross-correlations were conducted to create correlation coefficient (Pearson’s *r*) maps using AFNI 3dTcorr1D (Colon-Perez et al., 2016). Composite functional connectivity maps were generated using AFNI 3dTtest++ for cortical and subcortical seed regions to determine differences between male and female marmosets (*p* < 0.01). In addition, Pearson’s *r* coefficients per all ROI pairs were subjected to a voxel-wise z-transformation and exported for network analyses in MATLAB (MathWorks).

#### Network analyses

We calculated basic graph theory metrics to assess the topology of functional connectivity networks. Resting state fMRI data were analyzed using Brain Connectivity Toolbox for MATLAB (Rubinov and Sporns, 2010). Symmetrical connectivity graphs with a total 29,646 matrix entries were first organized in MATLAB (graph size = n(n − 1)/2, where n is the number of nodes represented in the graph, or 244 ROIs). The *z* score values of the graphs were thresholded at various levels (1%, 5%, 10%, 15%, 20%) for each subject to create matrices with equal densities before network metric assessments. Matrix *z* values were normalized by the highest *z* score, such that all matrices had edge weight values ranging from 0 to 1. Node strength (sum of edge weights), clustering coefficient (the degree to which nodes cluster together in groups), average shortest path length (the potential for communication between pairs of structures), modularity (the degree to which the network may be subdivided into clearly delineated groups or communities), and small worldness (the degree to which functional brain networks deviate from randomly connected networks) were calculated for weighted or unweighted graphs (Newman, 2003; Newman and Girvan, 2004; Boccaletti et al., 2006; Saramäki et al., 2007; Humphries and Gurney, 2008).

The small world index was determined by comparing marmoset functional connectivity networks to an average of ten null hypothesis networks per monkey (Watts and Strogatz, 1998). Thus, the ratio for clustering coefficients and path lengths of marmoset brain relative to null networks were calculated. The ratio of clustering coefficients is known as **γ**, which for a small world network is larger than 1 (Humphries and Gurney, 2008). The ratio of average path length is referred to as **λ**, which for a small world network is close to 1. The small world (sw) parameter is the ratio of **γ/λ**, with a sw > 1 indicative of small world topology (typical of real world networks) and sw ∼ 1 indicative of a random network (Erdös and Rényi, 1960). Brain networks were visualized using BrainNet (Xia et al., 2013). The 3D networks were generated with undirected edges weights *E*_undir_ ≥ 0.2. In these brain networks (or marmoset brain connectomes), the node size and color were scaled by the node strength, and edges were scaled by *z* scores.

## Results

### Cognitive tasks

#### SR learning

**Figure 3.**
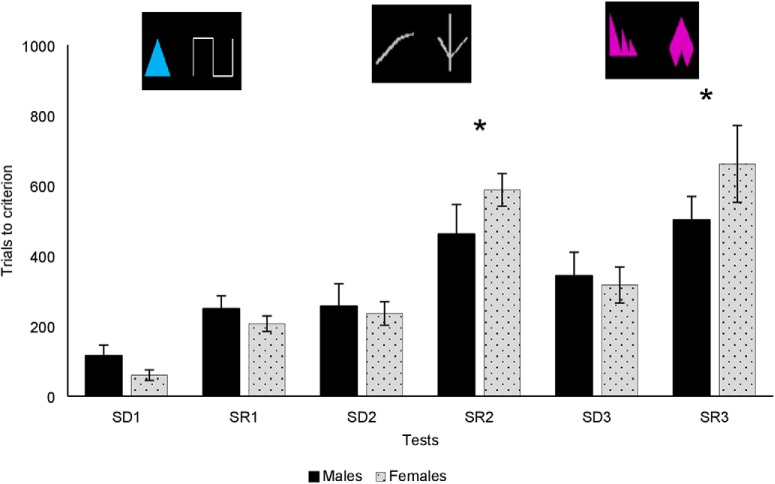
Mean trials to criterion ± SEM on the SR learning task in males and females; *significantly different at *p* < 0.05 (two tailed *t* test); examples of stimuli shown above.

Twenty-two monkeys (11 females, mean age = 5.05 years, SEM = 0.18; 11 males, mean age = 4.69, SEM = 0.14) completed the SR learning task (means for TTC, sessions, omissions, errors, and response latencies for each sex; Table 2). A significant interaction between sex and test type (*F*_(1,19)_ = 7.93, *p* = 0.01, partial η^2^ = 0.29) revealed that females needed more trials (M = 496.66, SEM = 53.17) than males (M = 401.22, SEM = 53.17) to reach criterion on the SRs, but not on the SDs (males: m = 235.16, SEM = 31.46, females: m = 213.33, SEM = 31.46; Fig. 3). A marginal sex × test type × pair number (*F*_(1.26,23.88)_ = 3.00, *p* = 0.088, partial η^2^ = 0.14) suggested that females were especially impaired for the more complex pairs, pair 2 and pair 3. RI was significantly greater in females than in males (*t*_(20)_ = −3.44, *p* < 0.01), reflecting poorer performance of the females in the reversals, relative to the pre-reversal discriminations.

**Table 2.** Mean TTC, sessions, omissions, errors, and response latencies for each sex

						Reversal learning							
		SD1		SR1		SD2		SR2		SD3		SR3	
	Sex	Mean	SEM	Mean	SEM	Mean	SEM	Mean	SEM	Mean	SEM	Mean	SEM
TTC	Male	114.71	28.13	245.68	35.06	256.11	61.04	466.18	81.08	334.64	62.76	491.79	64.43
	Female	73.28	11.50	203.85	23.42	241.71	32.75	581.92	50.46	325.00	55.78	704.21	113.37
Sessions	Male	7.91	2.22	17.27	2.76	11.00	3.00	19.55	3.05	13.45	2.49	22.27	4.64
	Female	3.64	0.68	15.82	2.72	11.00	1.46	21.91	2.51	12.18	2.27	27.09	5.15
Omissions	Male	139.91	49.01	371.82	92.81	189.64	66.85	350.82	98.29	219.45	58.02	465.64	175.87
	Female	57.36	10.43	355.91	122.89	149.00	40.18	265.36	60.76	171.00	53.12	386.55	172.72
Errors	Male	54.09	14.10	141.36	19.69	82.73	17.89	203.82	36.77	115.45	22.77	234.91	29.49
	Female	21.45	5.66	114.82	12.17	80.36	10.26	263.09	23.51	106.36	20.85	304.27	49.62
Response latencies (ms)	Male	3654.42	189.63	3340.06	136.84	2935.33	210.35	2847.08	188.39	2780.91	154.99	2714.02	204.92
	Female	3532.97	215.84	3382.48	155.75	2937.11	87.55	2778.18	147.96	2761.57	185.61	2747.72	178.55

						ID/ED set shifting							
		SD1		SR1		SD2		SR2		SD3		SR3	
	Sex	Mean	SEM	Mean	SEM	Mean	SEM	Mean	SEM	Mean	SEM	Mean	SEM
TTC	Male	193.47	81.81	673.33	155.91	411.33	73.60	458.52	97.93	789.53	180.24	1593.51	230.80
	Female	409.77	68.42	1094.87	130.38	597.07	61.55	785.33	81.90	679.43	150.73	1132.65	192.42
Sessions	Male	14.30	4.14	38.60	9.01	17.78	3.60	29.88	10.67	30.86	13.09	48.33	16.39
	Female	14.27	2.22	36.91	4.75	26.18	5.05	29.18	5.07	20.90	5.34	33.70	8.28
Omissions	Male	134.27	104.44	261.93	124.83	99.38	102.53	494.05	175.71	407.92	152.46	483.29	134.51
	Female	221.91	87.35	301.85	104.39	303.94	85.75	262.56	146.94	202.56	127.50	335.40	112.49
Errors	Male	49.88	32.74	312.72	75.41	154.31	31.04	215.45	49.25	294.30	67.28	608.58	123.24
	Female	145.68	27.38	513.80	63.08	233.48	25.96	377.79	41.18	246.99	56.27	533.39	103.06
Response latencies (ms)	Male	2776.09	330.47	2242.17	228.37	2255.34	208.17	2330.28	191.18	2315.74	162.22	1972.86	148.37
	Female	2322.95	330.47	2297.16	191.07	2379.70	174.16	2333.36	159.97	2348.26	135.73	2269.30	124.14

#### Errors to criterion

A significant effect of test type (*F*_(1,20)_ = 120.80, *p* < 0.001, partial η^2^ = 0.86) indicates that animals made significantly more errors during SR trials than during SD trials. A marginal sex × test type effect (*F*_(1,20)_ = 4.02, *p* = 0.059, partial η^2^ = 0.17) indicates that effect of test type was larger in females than in males. A follow-up ANOVA was performed examining only the more complex pairs 2 and 3, on which females needed more trials to reach criterion. When pair 1 was removed from the analysis, a significant sex × test type interaction was found (*F*_(1,20)_ = 6.14, *p* = 0.02, partial η^2^ = 0.24) again indicating that females were more affected by the SR trials in terms of errors than males.

#### Omissions and RTs

Monkeys omitted more trials during SRs (m = 366.02, SE = 64.59) than during SDs (m = 154.39, SEM = 25.17), as indicated by a significant main effect of test type (*F*_(1,19)_ = 5.87, *p* = 0.03, partial η^2^ = 0.24). There was also a significant effect of test number (*F*_(2,38)_ = 3.19, *p* = 0.05, partial η^2^ = 0.14), indicating that monkeys omitted more trials for pair 3 (m = 463.91, SEM = 50.01) and 2 (m = 386.48, SEM = 36.88) than pair 1 (m = 159.39, SEM = 15.48). Importantly, Sex did not affect the omissions (*F*_(1,19)_ = 0.48, *p* = 0.50, partial η^2^ = 0.02) and there were no significant sex × test type (*F*_(1,19)_ = 0.01, *p* = 0.91, partial η^2^ = 0.001) or sex × test number (*F*_(1,38)_ = 0.007, *p* = 0.99, partial η^2^ < 0.001) interactions.

In terms of RT, there were no significant effects of sex (*F*_(1,20)_ = 0.02, *p* = 0.89, partial η^2^ = 0.001), sex × test number (*F*_(1,20)_ = 0.03, *p* = 0.972, partial η^2^ = 0.001), sex × test type (*F*_(1,20)_ = 0.13, *p* = 0.72, partial η^2^ = 0.006), or sex × test number × test type (*F*_(1,20)_ = 0.4, *p* = 0.67, partial η^2^ = 0.02).

#### ID/ED

**Figure 4.**
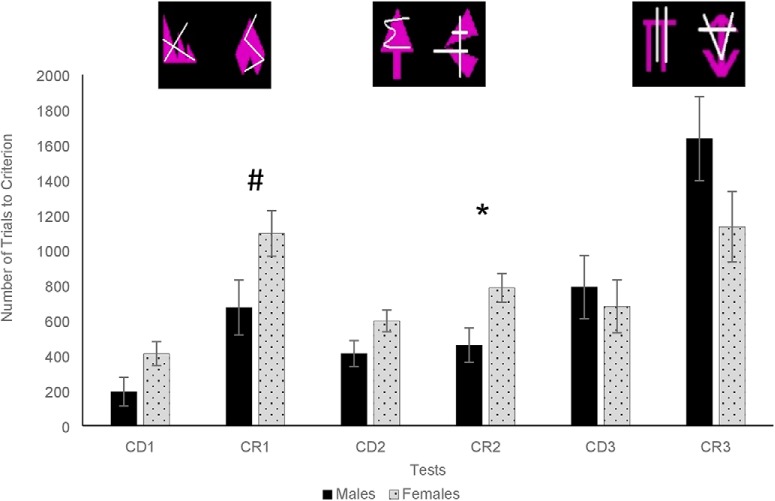
Mean trials to criterion ± SEM on the ID/ED in males and females; *significantly different at *p* < 0.05 (two tailed *t* test; # indicates marginal significance, *p* = 0.057); examples of stimuli shown above.

Seventeen out of the 22 original marmosets completed the ID/ED (10 females, mean age = 5.10 years, SD = 0.71; 7 males, mean age = 4.97 years, SD = 0.32, means for TTC, sessions, omissions, errors, and response latencies for each sex; Table 2). A significant test type × pair number interaction (*F*_(2,28)_ = 8.11, *p* = 0.002, partial η^2^ = 0.37) indicated that animals needed significantly more trials for CRs on pair 1 and pair 3. A significant interaction between sex and pair number (*F*_(2,28)_ = 3.84, *p* = 0.03, partial η^2^ = 0.22) revealed that females needed more trials to reach criterion than males on pair 1 (females: m = 752.32, SEM = 88.81; males: m = 433.4, SEM = 106.2; *t*_(15)_ = 2.37, *p* = 0.03) and pair 2 (females: m = 691.20, SEM = 53.07; males: m = 434.93, SEM = 63.45, *t*_(15)_ = 2.49, *p* = 0.03) but not on pair 3 (females: m = 906.04, SEM = 161.32; males: m = 1212.46, SEM = 192.90, *t*_(15)_ = 0.94, *p* = 0.36). Finally, a marginal sex × test type × pair number (*F*_(2,28)_ = 2.58, *p* = 0.093, partial η^2^ = 0.16) suggested that females were particularly impaired on CR2 (ID reversal; females: m = 785.33, SEM = 81.90; males: m = 458.52, SEM = 97.93, *p* = 0.02; Fig. 4), and tended to perform more poorly than males on CR1 (compound reversal; females: m = 1094.87, SEM = 130.38; males: m = 673.33, SEM = 155.91, *p* = 0.057).

#### Errors to criterion

There was a significant effect of test type (*F*_(1,14)_ = 64.37, *p* < 0.001, partial η^2^ = 0.82) with animals making more errors during the CRs than the CDs. There was also a significant effect of pair number (*F*_(2,28)_ = 21.17, *p* < 0.001, partial η^2^ = 0.60), with animals making more errors on pair 3 (ED shift/reversal) than on pair 1 (compound discrimination/reversal) or pair 2 (ID shift/reversal). The effect of sex (*F*_(1,14)_ = 1.91, *p* = 0.12, partial η^2^ = 0.16), sex × test type (*F*_(1,14)_ = 0.78, *p* = 0.39, partial η^2^ = 0.05), sex × pair number (*F*_(2,28)_ = 2.35, *p* = 0.11, partial η^2^ = 0.14), or sex × pair number × test type interactions (*F*_(2,28)_ = 0.40, *p* = 0.68, partial η^2^ = 0.03) were not significant.

#### Omissions and RTs

There was a significant main effect of test type (*F*_(1,14)_ = 9.45, *p* = 0.008, partial η^2^ = 0.40) indicating monkeys omitted more trials on CRs (m = 356.51, SEM = 68.25) than on CDs (m = 228.33, SEM = 52.76). There was also a significant effect of pair number (*F*_(2,28)_ = 6.47, *p* = 0.005 partial η^2^ = 0.32) with animals omitting more trials on pair 3 (m = 357.29, SEM = 68.37) than on pair 1 (m = 229.99, SEM = 50.73). Sex had no effect on omissions (*F*_(1,14)_ = 0.14, *p* = 0.72, partial η^2^ = 0.01), and there were no significant sex × test type (*F*_(1,14)_ = 2.77, *p* = 0.12, partial η^2^ = 0.17) or sex × pair number (*F*_(1,14)_ = 2.23, *p* = 0.13, partial η^2^ = 0.14) interactions.

In terms of RT, there were no significant effects of sex (*F*_(1,14)_ = 0.012, *p* = 0.91, partial η^2^ = 0.001) and no significant sex × pair number interactions (*F*_(2,28)_ = 2.05, *p* = 0.15, partial η^2^ = 0.13). Unlike RT for the reversals, a marginally significant sex × test type interaction was found (*F*_(1,14)_ = 3.33, *p* = 0.09, partial η^2^ = 0.19). Follow-up comparisons indicated that males had significantly longer RT on initial discriminations (m = 2458.72 ms, SEM = 186.22 ms) than on reversal trials (m = 2154.47 ms, SEM = 174.95 ms, *p* = 0.02). Importantly, males and females did not significantly differ on RT on the initial discriminations (*p* = 0.65) or the reversal trials (*p* = 0.49). Finally, there were no significant sex × pair number × test type interactions (*F*_(2,28)_ = 0.65, *p* = 0.53, partial η^2^ = 0.04).

### Social separation task

#### Behavior

**Figure 5.**
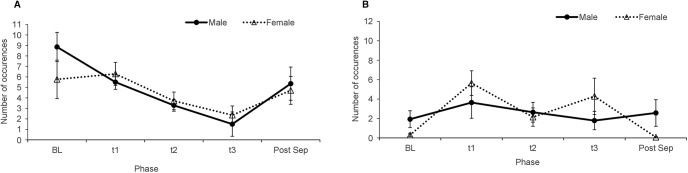
Behaviors before, during, and after separation (means ± SEM). ***A***, Calm locomotion. ***B***, Agitated locomotion.

Twenty-eight monkeys (14 females, mean age = 4.94 years, SD = 0.68; 14 males, mean age = 4.73 years, SD = 0.52) completed the social separation task. For agitated locomotion, there was a marginally significant sex × test phase interaction (*F*_(2,28)_ = 3.04, *p* = 0.06, partial η^2^ = 0.18). Follow-up *t* tests showed that females exhibited greater agitated locomotion during Sep than BL (*p* = 0.02) or Post-Sep (*p* = 0.06). Males’ agitated locomotor behavior was unaffected by the social separation (all *p*s > 0.05; Fig. 5*B*). Sex did not affect calm locomotion, but there was an effect of test phase (*F*_(2,28)_ = 4.86, *p* = 0.015, partial η^2^ = 0.26), with a decrease in calm locomotion from BL to Sep (*p* = 0.022) in both sexes (Fig. 5*A*).

#### Cortisol levels

**Figure 6.**
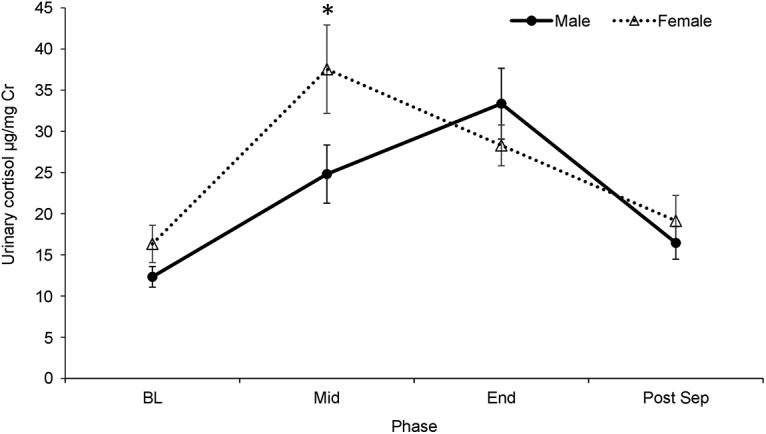
Urinary cortisol (mean ± SEM) before, during, and the morning following separation in males and females; *significantly different at *p* < 0.05 (two tailed *t* test).

Test phase had a significant effect on urinary cortisol levels (*F*_(3,30)_ = 9.63, *p* < 0.001, partial η^2^ = 0.49), with an increase in cortisol from BL to t2 (*p* = 0.003) and from BL to t3 (*p* = 0.006) and a return to BL levels of cortisol by the Post-Sep phase (*p* = 0.06; Fig. 6). To further investigate the relationship between sex and test phase, paired samples *t* tests were used to compare BL to t2, t3, and Post-Sep cortisol levels in females and males. A Bonferroni correction was used to correct for multiple comparisons. For females, cortisol levels significantly increased from BL to t2 (*t*_(5)_ = 3.81, *p* = 0.013) and t3 (*t*_(9)_ = 3.39, *p* = 0.008), but returned to BL levels by the Post-Sep cortisol measurement (*t*_(11)_ = 0.77, *p* = 0.459). In males, the increase in cortisol was delayed, with levels being similar to BL at t2 (*t*_(6)_ = 2.04, *p* = 0.087), significantly increased from BL at t3 (*t*_(13)_ = 4.77, *p* < 0.001) and not significantly different from BL at Post-Sep (*t*_(13)_ = 1.82, *p* = 0.092).

#### Stress and cognitive interactions

RI was not significantly correlated with change in cortisol from BL to t2 (*r*_(8)_ = –0.004, *p* = 0.99), or change in cortisol from BL to t3 (*r*_(18)_ = –0.12, *p* = 0.61). RI was also not significantly correlated with change in agitated locomotion from BL to t1 (*r*_(19)_ = 0.22, *p* = 0.35), from BL to t2 (*r*_(19)_ = 0.12, *p* = 0.60), or from BL to t3 (*r*_(19)_ = 0.25, *p* = 0.27). Interestingly, independent of sex, RI was significantly correlated with basal cortisol (*r*_(20)_ = 0.47, *p* = 0.026).

### Hill and valley task

Twenty-one monkeys (11 females and 10 males) completed the hill and valley task. The strength of the lateral bias (HI) did not differ between left and right handed individuals (independent *t* test, *t*_(13)_ = −1.41, *p* = 0.18). We examined the effects of Sex and Hand Use on the latencies to complete the tests as well as test scores, with HI as a covariate. For the latencies, there were no significant effects of hand use (*F*_(1,18)_ = 0.19, *p* = 0.67, partial η^2^ = 0.01), sex (*F*_(1,18)_ = 0.12, *p* = 0.73, partial η^2^ = 0.007), and no significant sex × hand use interaction (*F*_(1,18)_ = 0.218, *p* = 0.64, partial η^2^ = 0.012). For the score, there were no significant effects of hand use (*F*_(1,18)_ = 0.31, *p* = 0.59, partial η^2^ = 0.02), sex (*F*_(1,18)_ = 0.45, *p* = 0.51, partial η^2^ = 0.02), and no significant sex × hand use interaction (*F*_(1,18)_ = 2.79, *p* = 0.11, partial η^2^ = 0.31).

### rsFC

**Figure 7.**
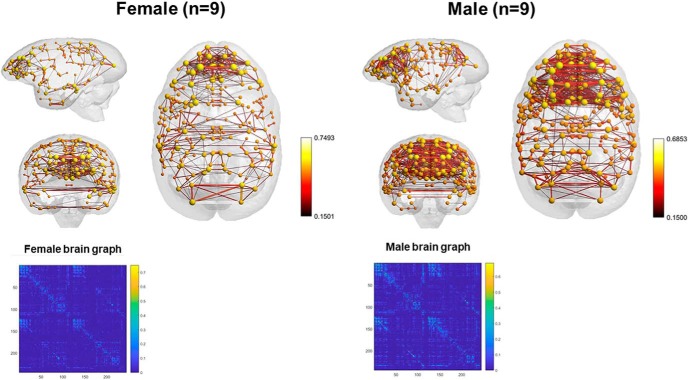
3D functional network maps of the marmoset monkey brain display increased clustering in the males relative to females. Shown are sagittal, coronal, and axial views of nodes (spheres) and edges (connecting lines) overlaid onto a 3D atlas map shell. Maps are thresholded at z > 0.2 and maps represent the top 10% of connections (density k = 0.10). The matrices below are mean functional connectivity matrices for male and female brains and their randomly rewired versions with the same density and edge weights and randomly assigned connections.

**Figure 8.**
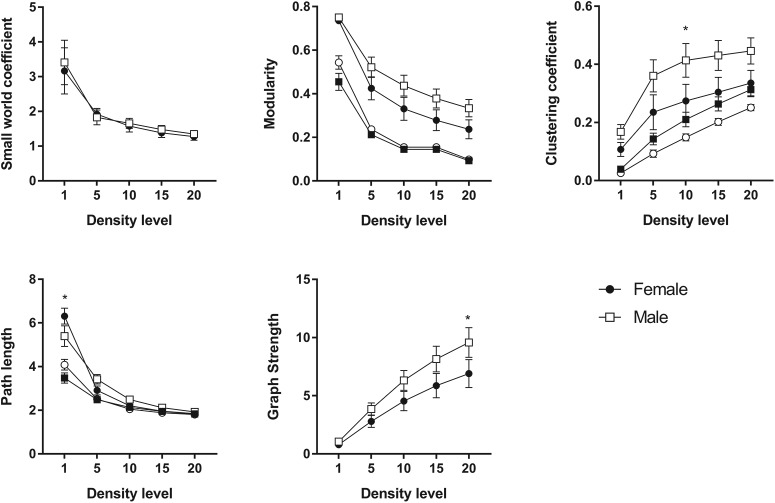
Functional network metrics indicate that males have a greater clustering coefficient than females; *significantly different at *p* < 0.05 (two tailed *t* test). Filled circles represent female data, and empty squares represent males. Empty circles and filled squares represent the same metrics calculated for random networks with the same density and edge weights.

**Figure 9.**
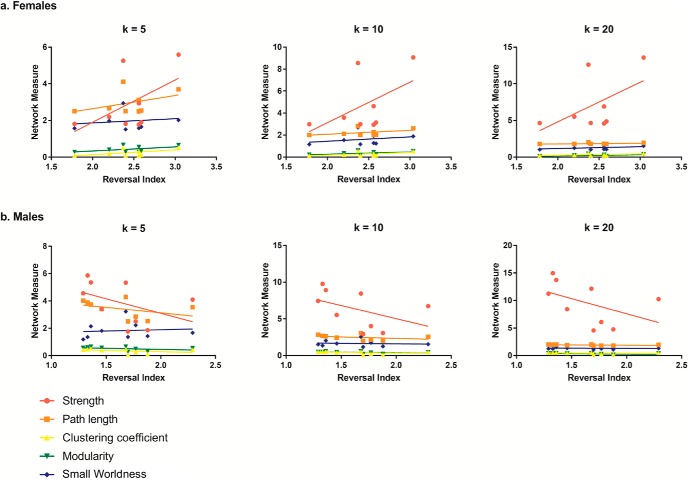
Functional network strength is positively correlated with cognitive performance in females and negatively correlated in males. ***A***, Linear regression between RI and several network measures (strength, path length, clustering, modularity, small worldness) indicates a positive correlation between only node strength and RI. ***B***, Similar to females, only node strength covaries with RI values. However, in males, this is observed as a negative correlation; k values represent different connectivity density thresholds.

**Figure 10.**
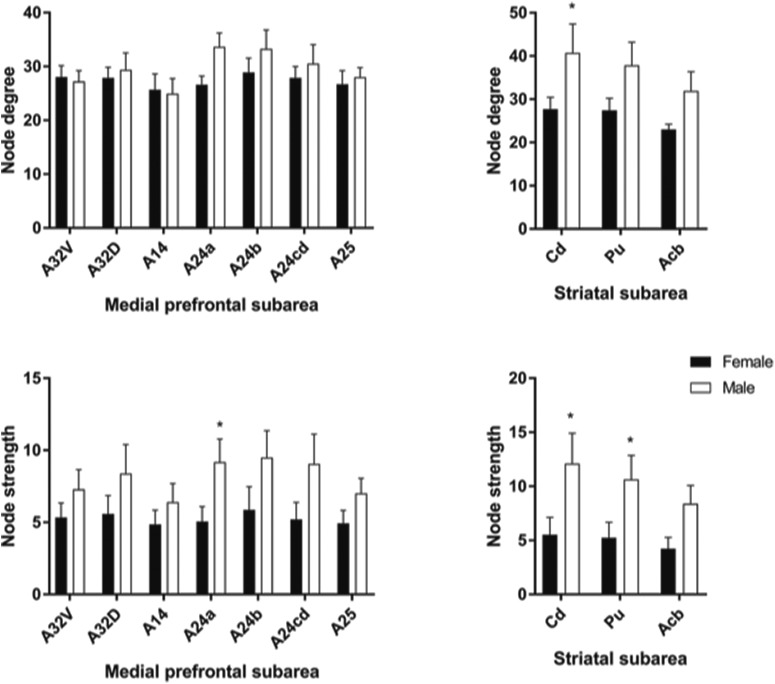
Medial prefrontal cortex (area 24) and caudate nucleus exhibit greater node strength in male marmosets compared to females; *significantly different at *p* < 0.05 (two tailed *t* test).

**Figure 11.**
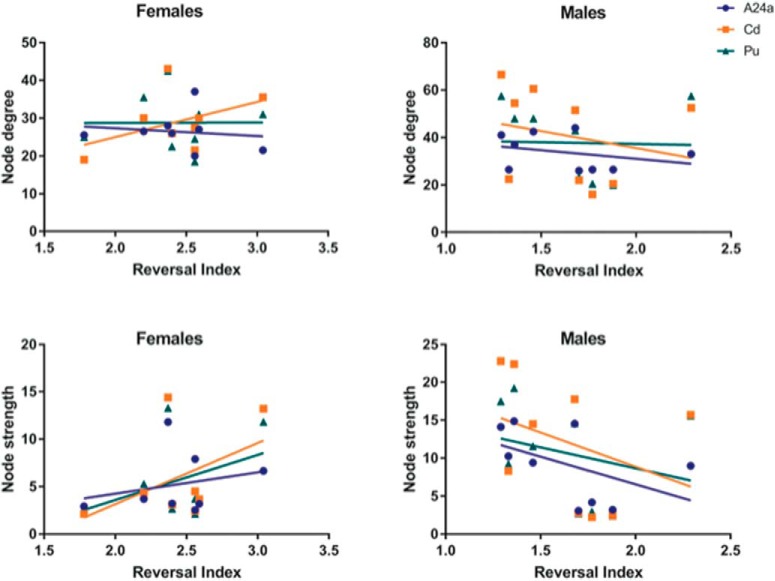
Functional network strength (and not node degree) in medial prefrontal cortex and caudate nucleus is positively correlated with cognitive reversal learning in females and negatively correlated in males.

**Figure 12.**
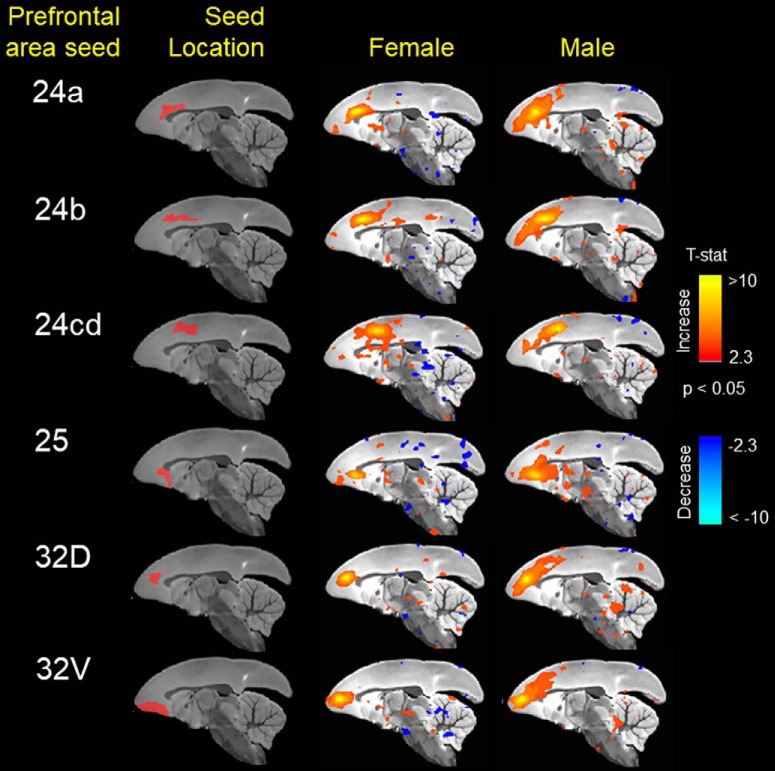
Seed based functional connectivity in various medial prefrontal cortex subdivisions indicates that males have greater functional connectivity with these regions than females. Maps represent mean functional connectivity across all animals within each group, thresholded by statistical *t* values (*t* > 2.3, *p* < 0.05).

**Figure 13.**
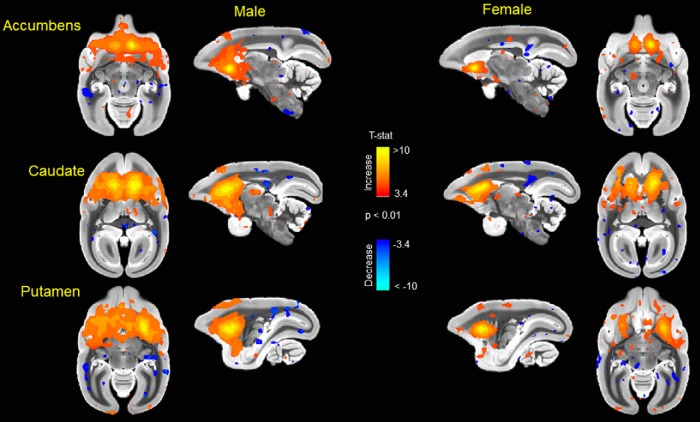
Seed based functional connectivity in caudate nucleus, putamen, and nucleus accumbens indicates that males have greater functional connectivity with these regions than females. Maps represent mean functional connectivity across all animals within each group, thresholded by statistical *t* values (*t* > 2.3, *p* < 0.05).

**Figure 14.**
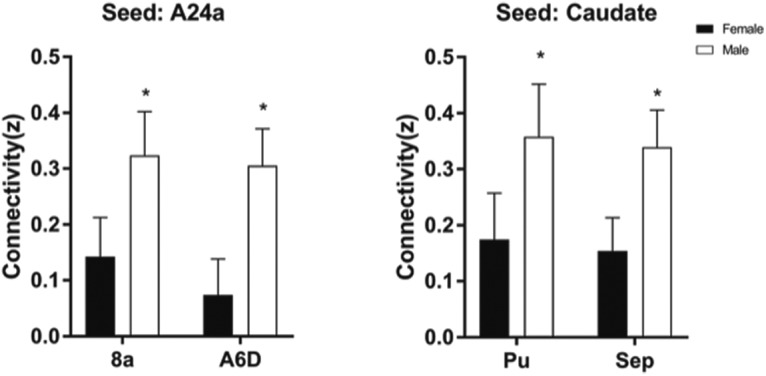
Greater functional connectivity in frontal cortical and striatal circuits of male marmosets compared to females. Seed regions are indicated above the plots; *significantly different at *p* < 0.05 (two tailed *t* test). Area 8a is located in the dorsolateral prefrontal cortex and A6D in the premotor cortex. Pu, putamen; Sep, septum.

Animals with cognitive data (nine female, mean age = 6.12 years, SD = 0.73; nine male, mean age = 5.88, SD = 0.57 years) were imaged, all within three months of cognitive testing. 3D functional network maps of the marmoset monkey brain revealed higher clustering in the males relative to females (Fig. 7), and functional network metrics indicated a greater clustering coefficient in males than females (*p* < 0.05; Fig. 8). Network node strength was positively correlated with the RI in females and negatively correlated in males, with a greater RIs reflecting poorer reversal performance. Linear regression between RI and several network measures (strength, path length, clustering, modularity, small worldness) indicated a positive correlation between node strength and RI in females, suggesting that greater node strength is associated with worse reversal performance in females. Similar to females, only node strength covaried with RI values in males, however, this correlation was negative (Fig. 9), indicating that greater node strength in males is associated with better reversal performance. Node strength in the medial prefrontal cortex (area 24) and the caudate nucleus was greater in male marmosets (*p* < 0.05; Fig. 10). Functional network strength (but not node degree) in the medial prefrontal cortex and the caudate nucleus was positively correlated with RI in females and negatively correlated in males (Fig. 11). Seed based functional connectivity values in multiple medial prefrontal cortex subdivisions (*p* < 0.05; Figs. 12, 14) and the caudate, putamen, and NAc (*p* < 0.05; Figs. 13, 14) indicate that males have greater functional connectivity with these regions than females.

## Discussion

We tested female and male marmosets on two executive function tasks: SR learning, a measure of cognitive flexibility, and the ID/ED, a measure of attentional set shifting. In SR learning, females performed more poorly than males in acquiring the reversals, especially for pair 2 and pair 3, which used stimuli (two shapes or two lines) that were more difficult to differentiate than those of pair 1 (shape vs line), as evidenced by an increase in both the number of trials necessary to reach learning criteria and the number of errors. Consistent with these findings, females also needed more trials to reach learning criterion on the reversal portions of the ID/ED, particularly on pair 1 and pair 2 reversals which required them to perform reversals without a change in attentional set. However, females performed similar to males on the ID or ED shifts. These findings point to a deficit of females specific to reversal learning, independent of a deficit in attentional set shifting. We ruled out motivation as a main contributor of this sex difference, as there was no sex difference in the number of trials the animals omitted or RT. Additionally, monkeys’ motor skills in the hill and valley were similar between males and females, thus it is unlikely that sex differences in cognitive performance were due to differences in motor ability.

We found a significant female deficit on reversal learning. This female deficit was specific to reversal acquisition and to the pairs that were most difficult to discriminate because the stimuli shared similar features (e.g., two lines of the same color). This finding is in agreement with human literature, which finds a male advantage in reversal learning in both children (Overman, 2004) and adults (Evans and Hampson, 2015). Interestingly, no sex difference was found in the first pair of stimuli, which involved discriminating between two stimuli with clearly different features, a shape and a line of different colors. This suggests that sex differences in reversal learning are sensitive to perceptual complexity, with the male advantage emerging only when the reinforcement contingencies involve discriminating among stimuli sharing similar features.

In the ID/ED task, marmosets needed more trials to acquire the ED than the ID. This finding is in agreement with previous studies, which have asserted that a shift within the same attentional set (ID shift) is acquired more rapidly than a shift to a new attentional set (ED shift; Owen et al., 1991; Dias et al., 1996). However, no sex difference was found in the ability to perform the ID or ED.

There is ample evidence that reversal learning and attentional set shifting are controlled by anatomically discrete brain regions. Two brain regions seem to be critical to reversal learning: the OFC and the striatum (for review, see Izquierdo et al., 2017). Functional imaging studies in humans have shown increased activation in the OFC during reversal learning paradigms (Nagahama et al., 2001; Cools et al., 2002; Ghahremani et al., 2010) and studies in NHPs have shown that lesions to the OFC cause disruptions in reversal, but not in the initial stimulus-reward associations (Izquierdo, 2004; Machado and Bachevalier, 2007). Dias et al. (1996) compared the performance of marmosets with OFC lesions, animals with lesions to the dorsolateral PFC (DLPFC), and sham-lesioned animals. Marmosets with OFC lesions showed impairments in reversal learning, but ED set shifting remained intact, while animals with DLPFC lesions showed opposite deficits. A similar pattern has been shown in rodent research, with lesions to the OFC impairing reversal learning but leaving attentional set shifting intact (McAlonan and Brown, 2003). In addition to the OFC, the striatum, which receives strong projections from the OFC, significantly contributes to reversal learning. Lesions to the medial striatum (Clarke et al., 2008) or dopaminergic depletion within the caudate (Clarke et al., 2011) cause impairments in reversal learning in the marmoset. Furthermore, a recent study found that infusion of the GABA_A_ agonist muscimol into the putamen led to impairments in reversal acquisition, while leaving SD unchanged (Jackson et al., 2019).

Based on these findings, it is likely that the observed sex difference in reversal acquisition reflects differences at the OFC/striatum level. The OFC has been implicated in the encoding of the associative value of a reward and is critical for updating this value for future decisions (Haber and Knutson, 2010). In contrast, the dorsal striatum mediates the acquisition and expression of habitual behavior, when the stimulus-response associations become automatized and less sensitive to the outcome (Fernandez-Ruiz et al., 2001; Miyachi et al., 2002; Graybiel and Grafton, 2015). This region of the striatum is also highly sensitive to estrogens (Di Paolo et al., 1985; Korol et al., 2004; Shams et al., 2016). In a prior study, we reported that estradiol (E2) replacement impairs reversal acquisition in ovariectomized female marmosets (Lacreuse et al., 2014), consistent with a detrimental effect of E2 on the dopaminergic striatal system. A recent study demonstrated that female rats engage in habitual behavior more rapidly than male rats during operant responding (Schoenberg et al., 2019). Based on these findings and the literature reviewed above, one interpretation of our results is that female marmosets may engage in habitual behavior earlier and/or to a greater extent than male marmosets while learning stimulus-response contingencies, impairing their ability to flexibly respond to new contingencies during reversal. The female impairment is most likely driven by effects of estrogens on the striatal dopaminergic system. Accordingly, one would expect reversal learning performance to vary with cycling endogenous E2 levels in female marmosets, as found for other striatal-dependent tasks in rodents (Becker et al., 1987). The specific mechanisms underlying these effects will have to be determined in future studies.

We examined whether stress reactivity to temporary social separation differed between males and females. While both sexes responded to the social stressor with decreased calm locomotion and an increase in cortisol, females, but not males, exhibited a significant increase in agitated locomotion during the stressor compared to baseline. In addition, the increase in cortisol levels occurred earlier in the separation for females than males. The significance of these findings is not entirely clear, but could reflect a delayed response to social stress in males. Basal cortisol was positively correlated with RI, indicating that increased basal cortisol was associated with poorer reversal performance for both sexes. This finding, consistent with human data (Kalmijn et al., 1998; Lee et al., 2007; Comijs et al., 2010), suggests that basal cortisol could be a marker for future age-related cognitive decline (Greendale et al., 2000; Beluche et al., 2010; Ennis et al., 2017; Pietrzak et al., 2017). Our ongoing longitudinal studies will determine the validity of this hypothesis.

Marmosets exhibit diurnal fluctuations in cortisol, with peak levels in the morning (9 − 10 A.M.) and decreasing throughout the day (Smith and French, 1997; Cross et al., 2004). In our animals, peak cortisol for females occurred at between 12 and 2 P.M. and between 2 and 4 P.M. for males, indicating that the pattern of cortisol increase was likely due to social separation paradigm and not attributable to normal diurnal cortisol fluctuations.

Important novel findings from the neuroimaging work indicate that (1) rsFC also revealed significant sex differences in connectivity patterns and activity strength and (2) rsFC patterns were differentially related to cognitive flexibility in males versus females. Specifically, male brains exhibited higher clustering of functional networks than females, had greater node strength in the medial PFC and the caudate nucleus, and greater functional connectivity than females in frontal cortical and striatal circuits. Interestingly, network strength was associated with better reversal performance in males, but worse reversal performance in females.

Prefrontal cortical circuits and striatal circuits have been identified as regions critical for reversal learning in marmosets (Clarke et al., 2005; Rygula et al., 2010; Jackson et al., 2019) with several neurotransmitters, including serotonin (Clarke et al., 2005), dopamine (Clarke et al., 2011), GABA (Jackson et al., 2019), and glutamate + glutamine Glx (Lacreuse et al., 2018) influencing cognitive performance. All these neurotransmitters are influenced by sex hormones (Barth et al., 2015) and at least one study reported a sex-dependent effect of Glx on reversal learning performance in marmosets (Lacreuse et al., 2018). In addition, these neurotransmitters (Lurie et al., 2018) as well as sex hormones (Arélin et al., 2015) also influence the architecture of functional connectivity networks. Human studies have suggested that sex differences in cognition are represented at the neural level by sex differences in the brain connectome (Gong et al., 2011; Ingalhalikar et al., 2014; Satterthwaite et al., 2015; Tunç et al., 2016; Ritchie et al., 2018). However, some data have been inconsistent (Satterthwaite et al., 2015 vs Tunç et al., 2016) and criticized for over-interpretation (Joel and Tarrasch, 2014). Animal studies, which minimize sociocultural influences on network activity and cognitive performance, may help clarify these relationships. It is important to note that the relationship between reversal learning and rsFC is correlational, and as such, causation cannot be asserted with our current data. However, as resting state network activation has been shown to reflect task-evoked activity in multiple studies (Mennes et al., 2010; Kannurpatti et al., 2012; Zou et al., 2013), it is likely that the male pattern of connectivity in our study represents a pattern that is more advantageous for reversal learning. Future studies should determine the potential role of sex hormones and neurotransmitters in shaping the functional connectome in males and females.

In summary, we found that female marmosets had poorer reversal learning relative to males. rsFC analyses revealed substantial sex differences in cognitive networks, with differences in both overall neural network metrics and specific regions, including the prefrontal cortex, caudate, putamen, and nucleus accumbens. Sex-dependent correlations between reversal learning and neural connectivity measures suggest that to sex-dependent patterns of resting brain networks may contribute to the sex difference in reversal learning. Although our data are correlational and cannot determine causal effects, they are consistent with human resting state data in supporting the idea that sex differences in cognitive performance have identifiable intrinsic neural correlates (de Lacy et al., 2019). Because of its relatively short lifespan, the marmoset should be particularly helpful in furthering our understanding of the dynamics of sex differences in cognition and associated brain networks across the lifespan.

## Synthesis

Reviewing Editor: Daniela Schiller, Mount Sinai School of Medicine Decisions are customarily a result of the Reviewing Editor and the peer reviewers coming together and discussing their recommendations until a consensus is reached. When revisions are invited, a fact-based synthesis statement explaining their decision and outlining what is needed to prepare a revision will be listed below. Note: If this manuscript was transferred from JNeurosci and a decision was made to accept the manuscript without peer review, a brief statement to this effect will instead be what is listed below.

Overall, the revision was less responsive than it could have been. Before returning the revised manuscript to the reviewers, please address the following issues:

General comment: the tenuous link between resting-state data and behavior deters mechanistic insight. A possible way to address this issue with the current data is to focus on a set of regions relevant to reversal learning and make a priori predictions. The current text indicates “122 bilateral placed seed regions included for 244 total ROI” (line 301) without specifying where those ROIs are and why were they selected. A more targeted analysis would strengthen the brain-behavior link. The focus on specific ROIs and the a priori predictions should be specified in the introduction.

Specific comments

For reviewer 1:

- Point 1: Information on “average number of trials per task, per session, number of sessions aborted, total number of trials per animal” seems to be missing

- Point 2: Please add this information to the methods section of the manuscript

- Point 4: Please give more information on how this was tested (ideally with framewise displacement), provide stats and add it to the manuscript

- Minor point 2: please also add F-value and partial eta-squared values for all tests on line 352, 353, 376, 377

For reviewer 2:

- Point 1: Even though stimuli appeared anywhere on the screen, animals could still show positional bias, e.g. according to movement distance and such bias could translate into differences in trials to criterion. The reviewer also asks about accuracy scores, perseveration, latencies, and anatomical differences. While not all of these measures may be needed, it would be good to focus on those that help understand better how the reported sex differences (and possibly the discrepancy with Takemoto and colleagues, 2015) come about. In contrast to the behavioral findings, the exclusive use of the reversal index for the analysis of neuroimaging data precludes us from restricting the effects to reversal rather than discrimination. Finally, contrary to the rebuttal response, the authors seem to have collected anatomical images which would allow them to test whether the hypothesized volumetric differences in OFC and striatum exist in their sample and explain the findings.

- Point 3: as point 1

- Point 4: it remains unclear how exaggerated and relaxed gaits were defined exactly. Step size? Please be precise and indicate a quantitative criterion. This is important information for reproducibility. Also, either use “relaxed” or “calm” but do not mix if they refer to the same thing.

- Point 7: Please provide a clear rationale for the appropriateness of using resting state data and for the three months time point and the limitations of this approach in providing mechanistic insight (e.g., the need to rely on reverse inference and indirect evidence from prior research on possible role of these regions).

Line 41, please clarify why the percentages do not add up to 100, there might be a typo.

On line 282, “Figures 3.5a-d” is unclear

“Sex” should consistently be in capitals when used to denote a factor in an ANOVA (lines 378-384)

Overall, the cortisol part appears rather weak in that it does not control for time of day. Accordingly, we do not know whether the differences in cortisol level are really due to separation or to diurnal differences. Moreover, why should more stress (as indicated by higher baseline cortisol) be positively (rather than negatively) related to better reversal performance? Both of these points should be discussed and/or mentioned as limitation

Line 542: it seems to be an overstatement to conclude anything about general cognitive flexibility, given that there were no differences in set shifting. Such statements should be removed throughout
